# A balancing act: how interpreters affect the therapeutic alliance in psychotherapy with trauma-affected refugees—a qualitative study with therapists

**DOI:** 10.3389/fpsyg.2023.1175597

**Published:** 2023-05-16

**Authors:** Saskia Hanft-Robert, Laura Glahder Lindberg, Mike Mösko, Jessica Carlsson

**Affiliations:** ^1^Department of Medical Psychology, Center for Psychosocial Medicine, University Medical Center Hamburg-Eppendorf, Hamburg, Germany; ^2^Mental Health Center Ballerup, Copenhagen University Hospital – Mental Health Services Copenhagen, Copenhagen, Denmark; ^3^Department of Clinical Medicine, Faculty of Health and Medical Sciences, University of Copenhagen, Copenhagen, Denmark; ^4^Department of Applied Human Sciences, Magdeburg-Stendal University of Applied Sciences, Magdeburg, Germany

**Keywords:** therapeutic alliance, interpreter, trauma, refugees, psychotherapy

## Abstract

**Objective:**

The therapeutic alliance (TA) has the highest predictive value concerning the success of psychotherapy. The presented study aimed to explore how the presence of an interpreter affects the TA when working with trauma-affected refugees.

**Method:**

Semi-structured interviews were conducted with seven psychologists working in an outpatient clinic specialized in mental health care for migrant and refugee patients with trauma-related mental health problems in Denmark. Interviews were transcribed verbatim and analyzed using a structuring content analysis approach.

**Results:**

TA has been described as a dynamic therapist-interpreter-patient alliance triangle consisting of three distinct but highly intertwined and mutually influential dyadic alliances. Specific factors affecting the quality of the TA were identified, e.g., interpreter being emotionally attuned yet not overly involved; interpreter being barely visible yet present as a human being. Characteristics of trauma-affected refugee patients affecting the TA formation were also identified, e.g., a high level of personal distrust, different understandings of mental disorders and psychotherapy, stigmatization, perceptions of authorities.

**Conclusion:**

The presence of interpreters was perceived ambivalently and the formation of a good TA seems to be a balancing act. Based on the findings, recommendations for forming and maintaining a good TA in interpreter-mediated psychotherapy are provided.

## 1. Introduction

With 30.9% (87 million) of the international migrant population residing in Europe, Europe is the primary destination for international migrants (McAuliffe and Triandafyllidou, 2021). While some people leave their country of origin voluntarily for educational, professional or family purposes, others are forced to leave due to on-going conflicts and wars, increased political instability, persecution, poverty, violence or geophysical and climate-related disasters. By the end of 2021, 89.3 million forcibly displaced people were estimated by the UN Health Refugee Agency worldwide (UNHCR, 2022). 27.1 million of them were refugees fleeing mainly from the Syrian Arab Republic, Venezuela, Afghanistan, South Sudan and Myanmar (UNHCR, 2022). Due to pre-migration traumatic experiences and post-migration stress, refugees are considered a particularly vulnerable group that is more prone to mental disorders. Compared to people without refugee experience, they have a significantly higher prevalence of mental disorders with PTSD, depression and anxiety disorders being the most prominent ones (Turrini et al., 2017; Blackmore et al., 2020). Despite their high need for psychological support, refugees tend to underutilize mental health care services (Satinsky et al., 2019). Due to a lack of multilingual mental health care professionals (Mösko et al., 2013) and policies for the use of qualified interpreters (Solano and Huddleston, 2020), language barriers are considered one of the main obstacles for refugees accessing mental health care services and receiving appropriate treatment (Ohtani et al., 2015; Satinsky et al., 2019).

### 1.1. Working with interpreters in psychotherapy

While there are already some qualitative studies exploring general challenges and benefits of interpreter-mediated psychotherapy (IMP) (Bauer and Alegría, 2010; Hanft-Robert et al., 2018; Gryesten et al., 2021) or potential risks of untrained interpreters, such as patients’ friends, family members or multilingual staff (Flores, 2005; Karliner et al., 2007; Kilian et al., 2014), only a few studies measured the effectiveness of psychotherapy mediated by (qualified) interpreters quantitatively. Most of these studies focus on the treatment of trauma-affected refugee patients and compared interpreter-mediated Cognitive Behavioral Therapy (CBT) with CBT conducted without an interpreter. Almost all studies found that psychotherapy mediated by qualified interpreters can be as effective as psychotherapy conducted without an interpreter (Schulz et al., 2006; d’Ardenne et al., 2007; Brune et al., 2011). In contrast, a more recent study conducted by Sander et al. (2019) showed that in comparison to no use of qualified interpreter, the use of an qualified interpreter in CBT with trauma-affected refugee patients is associated with less improvement in mental health outcomes, such as PTSD, depression and anxiety symptoms. The authors argued, among other things, that the presence of an interpreter might affect the TA, which could be a possible reason for the poorer treatment outcomes.

### 1.2. The concept of therapeutic alliance

The TA is considered one of the most important mechanisms for change in psychotherapy (Bordin, 1979; Horvath and Luborsky, 1993; Ardito and Rabellino, 2011). Over the last decades it has been shown that a good TA is moderately but consistently associated with positive treatment outcomes across therapeutic orientations, patient characteristics, countries and outcome measures (Ardito and Rabellino, 2011; Flückiger et al., 2018). Historically, the concept of the TA has its roots in Freud’s transference theory (1912) and the assumption that as a result of the therapist’s supportive attitude, patients would project onto their therapist early images of people from whom they had been treated with affection (Freud, 1958; Horvath and Luborsky, 1993). Over the decades, however, the understanding of the therapeutic alliance evolved away from projection and transference toward a reality-based, non-neurotic and non-transferential therapeutic collaboration between patient and therapist (Horvath and Luborsky, 1993). One of the most commonly used definitions today, which can be applied beyond psychoanalytical approaches to any therapeutic orientation and is thus known as the pan-theoretical conceptualization (Horvath and Luborsky, 1993), was formulated by Bordin (1979). According to him, a good TA consists of three essential components: agreement on the goals of the therapy, agreement on the tasks, and the development of a personal bond between therapist and patient (Bordin, 1979). The latter component is fundamental for the development of the first two (Bordin, 1979; Ardito and Rabellino, 2011).

### 1.3. Therapeutic alliance in interpreter-mediated psychotherapy

A traditional therapeutic setting consists of a therapist and a patient, both sharing one (native) language and communicating verbally with each other. The formation of the TA depends solely on the interaction between the patient and therapist. Referring to Bordin (1979), both have to agree on the therapeutic goals and tasks and form a personal bond. However, if a third person, an interpreter, gets involved, the formation of an alliance seems to become more complex and complicated.

To date, only one study could be found that measured specifically the association between the use of interpreters and the TA. In a study with 458 Spanish-speaking patients in the U.S. Villalobos et al. (2016) could not find significant differences in the TA between interpreter-mediated mental health care consultations and consultations conducted with a bilingual practitioner. However, in additionally conducted qualitative interviews with patients with limited language proficiency (LLP), practitioners and interpreters, Villalobos et al. (2016) found that most practitioners and LLP patients would prefer a treatment without an interpreter. Bilingual practitioners described a better rapport and a stronger sense of collaboration when working without an interpreter. Reasons for LLP patients’ preference of treatment without an interpreter included a feeling of greater privacy, enhanced communication, and a stronger TA especially in terms of trust and mutual understanding. However, both considered interpreters to be an excellent alternative to LLP patients not being able to use services at all (Villalobos et al., 2016).

Similarly, other qualitative studies have shown that the presence of an interpreter is often perceived by therapists and patients as a double-edged sword in terms of the TA (Raval and Smith, 2003; Miller et al., 2005; Tribe and Thompson, 2009a; Hanft-Robert et al., 2018, 2021). A previous interview study exploring the perspective of trauma-affected refugee patients on the TA in IMP found that especially at the beginning of the therapy, the presence of an interpreter as a second unknown person as well as the perceived lack of professionalism were described as disruptive factors for building trust and sharing personal, intimate experiences. At the same time the interpreter was considered as necessary to convey words as well as emotions and to form an alliance with the therapist (Hanft-Robert et al., 2021). Interview studies with therapists demonstrated that they are often concerned that an interpreter will hinder the development of a personal bond with the patient and make it more difficult to use therapeutic techniques to foster trust and rapport, such as reflective listening and conveying empathy (Raval and Smith, 2003; Miller et al., 2005; Krystallidou et al., 2018). In contrast, a recent study conducted by Delizée and Michaux (2022) found that an interpreter can actively co-create a supportive alliance, promoting patient’s self-expression and thus strengthen the therapeutic work. In line, other studies have shown that the presence of an interpreter is also perceived as beneficial. For instance, if the concept of psychotherapy and mental disorders is not familiar and patients are afraid of stigmatization, the presence of an interpreter as a representative of the patients’ cultural and linguistic backgrounds can normalize the experience of psychotherapy (Miller et al., 2005; Tribe and Thompson, 2009b). In the literature, it is described that the interpreter can also act as a cultural mediator between the patient and the therapists (Westermeyer, 1993; Miller et al., 2005; Hunt and Swartz, 2017; Hanft-Robert et al., 2018). While the interpreter is often perceived by therapists as a beneficial source of cultural information (Hanft-Robert et al., 2018), it should be kept in mind that individual differences and heterogeneity exist also within cultural groups (Palmer, 2004). If the interpreter acts on his or her own cultural interpretations, beliefs, and perceptions that do not align with those of the patient, the therapeutic process may be compromised (Kaufert and Koolage, 1984; Kilian et al., 2014).

Although, the presence of an interpreter is perceived ambiguously, it is repeatedly emphasized that, just as in traditional dyadic therapy, a good TA is also a prerequisite for a successful IMP (Miller et al., 2005; Tribe and Thompson, 2009a; Hanft-Robert et al., 2021). However, studies examining this specific therapeutic mechanism in IMP are scarce. The presented study aimed to explore from the therapists’ perspective how the presence of an interpreter affects the TA when working with trauma-affected refugee patients. Based on the results, recommendations will be derived on how to form a good TA.

## 2. Materials and methods

In the current study, semi-structured interviews were conducted with psychologists working in an outpatient clinic specialized in mental health care for migrant and refugee patients with trauma-related mental health problems in Denmark. The reporting of methods is in accordance with the consolidated criteria for reporting qualitative research (COREQ) (Tong et al., 2007).

### 2.1. Participants and recruitment

Selection of participants was based on a purposive sampling approach (Marshall, 1996). Included in the study were participants (1) working as psychologists at the outpatient mental health care clinic and (2) having completed at least one course of IMP (a minimum of 10 therapy sessions) at this specific clinic with (3) trauma-affected refugee patients. The research project was presented to all psychologists who were working in the outpatient mental health care clinic at the time of the study at one of their weekly meetings. In addition, all of them were invited to participate in the study *via* e-mail. One psychologist did not wish to participate and two did not respond to the email. The remaining seven participants were provided with oral and written information in Danish about the study prior to the interview. All gave their written, informed consent to be interviewed and for the interview to be digitally audio recorded, transcribed and analyzed for the purpose of the study. The study was not reviewed by a research ethics committee. In Denmark no ethical approval is required for studies using questionnaires or interviews that do not involve human biological material.^1^

### 2.2. Interview guide

The interview guide for this study was based on a literature review and the research question. It was developed by SHR in close consultation with LGL and JC following Helfferich’s (2009) SPSS approach of collecting, reviewing, sorting and finally subsuming questions. The guide covered the following topics: importance of TA in general and specifically in interpreter-mediated psychotherapies with trauma-affected refugees, challenges and benefits of having an interpreter involved, factors that facilitate or complicate establishing a good TA. Since the aim of the study was to explore new aspects and get in-depth insights into the psychologists’ experiences, open-ended questions were asked which aimed to encourage interviewees’ self-reflection (e.g., “Could you tell me a little about how a good TA between you and the patient can be build?” and “How did you know that you and the patient had a good alliance?”). The concept of TA can be rather abstract and theoretical and difficult to grasp or describe verbally. Therefore, participants were also invited to give examples (e.g., “Can you give me an example where you perceived the presence of the interpreter affected the alliance between you and the patient negatively?”). The guide was pilot tested twice with clinical psychologist by SHR, no significant changes were made.

### 2.3. Data collection and transcription

The interviews were conducted between March and April 2022 by SHR in person and in a one-on-one setting in the participants’ offices at the outpatient clinic. All interviews were conducted in English. SHR had no previous relationship with the participants. All interviews were conducted using the semi-structured interview guide described above. The guide allowed to deviate from the pre-formulated questions and ask individual questions to explore new or unexpected aspects that arose during the interview. Additionally, participants completed a short questionnaire on sociodemographic data. The interviews lasted between 51 and 117 min. The interviews were digitally audio recorded and transcribed verbatim by SHR and a trained student research assistant. All transcripts were proofread by SHR and not returned to participants. In order to protect participants’ identity, any personal data that could lead to identification (e.g., names of participants, patients or interpreters) was deleted or changed. Participation in the study was voluntary and not remunerated.

### 2.4. Data analysis

Data analysis followed Kuckartz’ (2014) structuring content analysis approach. A combination of deductive and inductive coding was applied. Deductive categories were derived from the interview guide and were supplemented by inductive categories when new aspects emerged from the interview data during the coding process (Kuckartz, 2014). To ensure intersubjective comprehensibility and credibility (Creswell, 2013), three interviews were coded separately by LGL and SHR. Coders read the interviews to get familiar with the data. Text fragments ranging from a sentence to one or more paragraphs were allocated to categories (inductive or deductive), which were later summarized and combined into main and subcategories. Subsequently, the coded text fragments and assigned codes were discussed by LGL and SHR. SHR analyzed the remaining interviews in close consolation with LGL. Final results were discussed with JC and MM. Study participants did not provide feedback on the final results. Data was analyzed using MAXQDA 2020.

### 2.5. Researchers’ characteristics

Qualitative researchers are intimately involved in the research process and personal preconceptions cannot be completely avoided. Therefore, researchers should clarify their identity, credentials, occupation, gender, experience and training (Tong et al., 2007). SHR is a female psychologist (M.Sc.) and doctoral student. She has many years of experience in conducting semi-structured interviews and qualitative data analysis. LGL is a female MSc and PhD in Public Health and has considerable experience with qualitative research methods and the field of cultural psychiatry. MM is a male psychotherapist and professor for clinical psychology with comprehensive experience in qualitative research. JC is a female MD, a research associate professor at the University of Copenhagen and head of research at the outpatient mental health clinic. She has many years of experience in conducting and supervising research in mental health consequences of trauma in refugees and in transcultural psychiatry.

### 2.6. Sample

The sample consisted of seven psychologists (*n* = 6 female, *n* = 1 male) working at the outpatient mental health care clinic specialized in treatment of migrant and refugee patients with trauma-related mental health problems in Denmark. The participants were between 30 and 50 years old (*M* = 39 years). They had between 3.5 and 19 years of working experience as a psychologist (*M* = 10.6 years) and between 3.5 and 11 years of experience in conducting IMP with trauma-affected refugee and/or migrant patients (*M* = 7.9 years). All participants had their licence in Cognitive Behavioral Therapy (CBT). Two psychologists were additionally trained in Acceptance and Commitment Therapy (ACT) and one psychologist in Narrative Exposure Therapy. All study participants conducted interpreter-mediated therapy several times a week (range 2–9 times per week). The majority (*n* = 6) had never received explicit training for working with interpreters. However, one interviewee noted that working with interpreters is a focus of and an ongoing dialog within the clinic. One psychologist had participated in a 60-h course about working with interpreters. Two psychologist had a migration background themselves (European countries) and two had a second-generation migration background (Global South). Three participants had no first- or second-generation migration background.

## 3. Results

**Figure 1:**
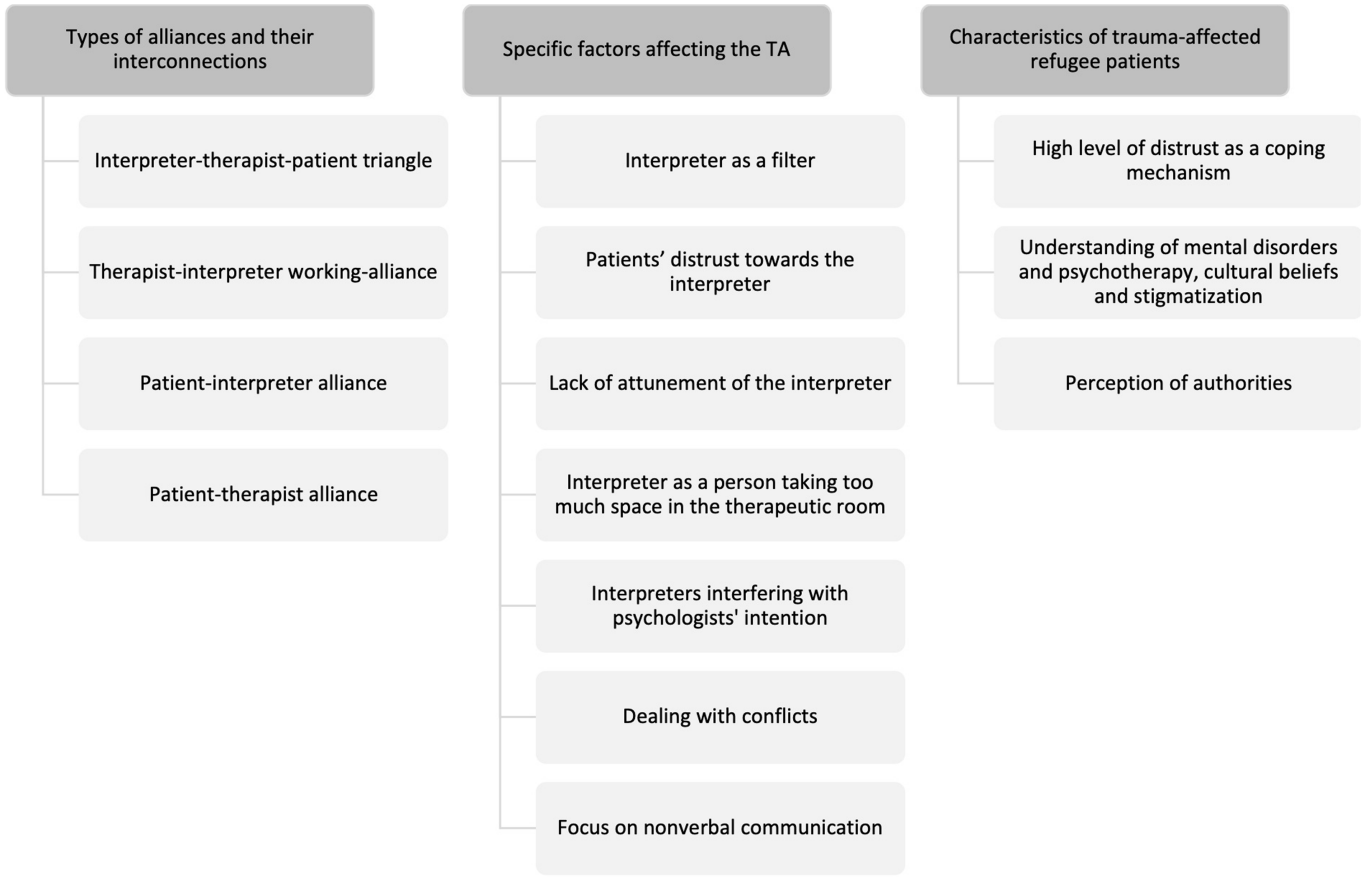
Identified main and subcategories regarding how interpreters affect the TA.

In total, 3 main categories with 14 subcategories and several sub-subcategories were identified. The first main category addresses the different types of alliances and their interconnections in IMP. It consists of 4 subcategories and several sub-subcategories. The second main category refers to factors positively or negatively affecting the alliance formation in IMP. It encapsulates 7 subcategories and several sub-subcategories. During the interviews it became evident that, in addition to the presence of an interpreter, certain characteristics of trauma-affected refugees must be taken into account in order to establish and maintain a good TA. Thus, the third main category comprises characteristics of trauma-affected refugee patients that were described as crucial for the TA. It consists of 3 subcategories, which in turn encapsulates several sub-subcategories (see Figure 1). Based on the results, recommendations are made on how to form a good TA (see Table 1).

**Table 1:** Therapists’ recommendations for forming a good TA in IMP based on the study results.

Therapist	Being aware that the interpreter is an active part of the alliance Creating a personal bond and agreement on tasks and goals of the therapy with patient and interpreter Attention to (distrust, ruptures and conflicts in) all three dyadic alliances Finding a balance between closeness and professional distance in the working alliance with the interpreter Use of qualified (professional) interpreters and recognition of interpreters’ professionalism Briefing the interpreter about therapeutic interventions and goals to avoid contradictory behavior on part of the interpreter Explain interpreters’ role and their obligation to adhere to professional ethical principles, such as confidentiality, impartiality and transparence Appropriate handling of conflicts during or after the session, depending on whether the conflict affects the patient and therapeutic process directly Focus on nonverbal behavior to strengthen the therapist-patient alliance, including body language (e.g., leaning a bit forward toward the patient), tone variation in the voice, having eye contact with the patient also when the interpreter is talking, active listening (saying “hmm,” nodding), and reacting with facial expressions toward the patient when the interpreter speaks Arrange seating position so that the patient and therapist have eye contact Awareness of that interpersonal trauma can prolong and complicate alliance formation and use of interventions focusing on strengthen the TA Cultural-sensitive psychoeducation: Exploring patients’ understanding of mental health disorders and psychotherapy/healing and creating a shared understanding (i.e., shared goals and tasks of the therapeutic process) Explaining the framework of psychotherapy Clarifying therapists’ role to the patient in contrast to other authorities
Interpreter	Shifting patients’ attention toward the therapist by eye movements or gentle hand gestures Continuous presence Sufficient language skills in both languages Adherence to professional ethical principles, such as confidentiality, impartiality, transparence, interpreting completely Emotional attunement by adapting the way of interpreting (e.g., tone of voice, volume, tempo, timing, body language) Finding balance in terms of personal and emotional involvement depending on the therapeutic process

### 3.1. Types of alliances and their interconnections

#### 3.1.1. Interpreter-therapist-patient triangle

The psychologists described that the TA cannot be thought of without the interpreter which, in turn, makes the development and maintenance of a good TA more complex and challenging: *“I do not really see it as me only with the patient […]. It’s like a triangulation, so they [the interpreters] are part of, you can say, the constellation, so they play a major role in me being able to build a good TA with the patient.” (IP06).*

The therapist emphasized that they need to be aware not only of their alliance with the patient but also of their working alliance with the interpreter as well as the patient-interpreter alliance. The desired goal is the development of a sense of teamwork between therapist, patient and interpreter.

#### 3.1.2. Therapist-interpreter working-alliance

All participants stressed the importance of having a good working alliance with the interpreter, which was described as being close, but not too close, as having a good balance between being friendly and professional with each other. This also includes mutual appreciation of each other’s professions and, especially on the part of psychologists, recognition of interpreters’ professionalism. The collaboration should include briefing and debriefing and allow mutual honest and constructive feedback. One psychologist pointed out that a good working alliance between the interpreter and therapist makes it possible to overcome irritations or ruptures due to misconduct on the part of the interpreter*: “She made a small correction of something I did because it was-, there was something cultural I missed in my intervention that she wanted me to be aware of […]. It did not put any harm to the alliance. […] Because she does not do it very often and I know her well. And I work with her and we have a good alliance. So it was just, you know, if she did that every time I would be so annoyed […]. So, you know, it’s very important to have a good alliance with the interpreter as well” (IP03).*

The participants stated that a good working alliance with the interpreter can give the patient a sense of a safety. Moreover, perceived ruptures or distrust in the working alliance can also affect the patient: *“If the patient can feel that I do not really trust the interpreter, then, it’s a rupture in the connection. Because we need that person. You know, so then there is distrust in the entire room. Which is, it’s not good.” (IP01).*

However, it was also noted that a too strong alliance between the psychologist and interpreter could make the patient feel excluded.

#### 3.1.3. Patient-interpreter alliance

The participants explained that the interpreter is often perceived as a person holding a safe space by the patient, as someone who can comfort them and, especially at the beginning of the therapy, facilitate the development of a trusting TA between the patient and the therapist. However, if the patient is permanently mainly attached to the interpreter, frustration and a sense of competition can arise. Psychologists described feeling excluded, superfluous, overlooked, powerless and incompetent. Some noted that the interpreter can help shifting the patients’ focus away from them and toward the therapist for example by using an eye or gentle hand gesture to indicate that the patient should look at the psychologist. One psychologist mentioned to disengage, when they have the feeling that there is no chance to build an alliance with the patient that is as strong as the interpreter-patient alliance. However, they also described that in some cases when there is such a strong alliance perceived between the interpreter and patient, they try to use it and work through that strong interpreter-patient alliance with the patien*t: “I think it would be like leaning back more. And accepting that the alliance is very much there. And that the person is receiving help through the interpreter. From the interpreter. It feels for the patient like it’s from the interpreter, but then I can remember – remind myself that it is through the interpreter […]. So, the change or therapeutic effect is sort of shifted on to whatever is going on between them. And that can be OK. That can be OK. It can work. And also be very mindful of it’s still my responsibility. That is not just the interpreter that is doing whatever or saying whatever. I’m also still the one who is actually leading the conversation. And structuring the conversation. Asking the questions.” (IP05).*

Another psychologist described that ruptures in the alliance between the patient and the interpreter, such as the interpreter coming late, laughing about something the patient says, being distracted while interpreting, breaking confidentiality, or interpreting incompletely, can also affect the therapist-patient alliance. These ruptures occur in a space where the therapist is not only physically present but also in charge of what is happening*: “Yeah, in some ways you are representing this place you know, and in some ways, if the rupture is happening in the relation to the interpreter, it’s still happening in your room. It’s still in the psychotherapeutic room that this is happening. So it’s impossible to-. I think it’s impossible that it does not in some way transfer, you know, contaminate the [therapist-patient] relationship.* “*(IP04).*

In contrast, one psychologist described that even if there are ruptures in the interpreter-patient alliance and a lack of trust toward the interpreter, there can be at the same time a strong trusting TA between the therapist and the patient. He/she described a situation where both, he/she and the patient were irritated by the interpreter. Although neither had a good alliance with the interpreter, there was a good alliance between them*: “I had a very good alliance with a patient even though there was an interpreter in the room, but not very good with the interpreter and she had not a very good one with the interpreter as well. But there was something between me and her [the patient] that understood, even though the interpreter wasn’t that good. She was quite irritating, the interpreter. And I think the patient also had that opinion. “(IP03).”*

#### 3.1.4. Patient-therapist alliance

All psychologists emphasized that, although an interpreter is involved, the TA between a therapist and a patient is still the focus. A clear communication of the role of the interpreter and therapist was described as crucial. However, the participants expressed that the patient-therapist alliance is dependent on a third person, which can be challenging. On the one hand, it was stated that interpreters can facilitate the development of the alliance between the therapist and patient, as they enable linguistic communication. On the other hand, interpreters can impede the alliance. Some psychologists mentioned that they try to talk to the patient directly every now and then, for example if the interpreter is late or has already left after therapy. If they feel that the patients’ language skills are reasonably sufficient, some therapists prefer to continue the therapy without an interpreter. One psychologist described how deciding to work without an interpreter changed the therapist-patient alliance significantly for the better*: “And she [the patient] was actually quite good at English and Danish. So after maybe first half of the whole therapeutic course we started doing our session without the interpreter. And that really did a massive change in the TA. That she [the interpreter] wasn’t there anymore. So, it wasn’t that she wasn’t nice or that she did not interpret correctly. She just had this presence that somehow disturbed the alliance between me and my patient. “(IP05).*

### 3.2. Specific factors affecting the therapeutic alliance

#### 3.2.1. Interpreter as a filter

Two psychologist pointed out that due to the language barrier the interpreter is the first person who gets the information from the patient and the also first chance to react to what the patient has said. The therapist, however, receives the (verbal) information with a delay which can lead to a feeling of distance in the therapist-patient alliance and in turn to a closeness in the interpreter-patient alliance, especially at the beginning of the therapy.: *“Here is the interpreter, the first person who gets the information that you have said. I, as psychologist, I’m left out, I do not know what they are talking about, but I can see this interaction and response from the interpreter. […] Generally it is easier to be a therapist when you do not have an interpreter. […] There is no filter, you get through with your message or your intervention directly and quickly to the patient.” (IP02).*

#### 3.2.2. Patients’ distrust toward the interpreter

All psychologist stated that the existence of mutual trust is the core of a good TA. The presence of an interpreter, however, can lead to mistrust on part of the patient for various reasons. Firstly, patients have to establish trust not only with one but with two strangers. This can be particularly challenging when taking into account that trauma-affected refugee patients generally have difficulties building trust. Secondly, the patients can perceive the interpreter as a representative of patients’ own culture who might judge them negatively based on cultural beliefs and norms. Thirdly, a suspected connection of the interpreter to the patients’ community can lead to distrust. Patients are worried that interpreters do not adhere to confidentiality and share sensitive information about them and the therapy within their community. Mistrust toward the interpreter can lead to the patient becoming less engaged in the therapy and even withholding therapy-relevant information*: „So, if I am in the position of the patient. I think that I would be worried about what the interpreter is going to think about me. I would be worried that I am being judged. I would be worried that the interpreter is going to talk in the community about me and my problems. Then I do not want to even engage in this therapy and open up. “(IP05).*

The participants named a number of factors that they believed could increase patients’ trust in the interpreter. This includes the continuous presence of the same interpreter; being impartial; having sufficient language skills and interpreting precisely and completely*: “I’m just thinking of one [case] where the patient was really happy about the interpreter too. And it wasn’t necessarily that the therapy had a big effect on the person, but I could just feel that there was a big trust from the patient both to me and the interpreter because the interpreter was so calm and so precise it made the patient feel really safe in the room […] and that she could express herself freely “(IP04).*

#### 3.2.3. Lack of attunement of the interpreter

Another challenge mentioned by all psychologists is when the interpreter is not appropriately attuned to them or the patient. All psychologists agreed that a good interpreter must sense the atmosphere, be emotionally attuned to what is happening in the room and be able to adapt the way of interpreting to it (e.g., tone of voice, volume, tempo, timing, body language). A lack of attunement can lead to irritation on the part of the patient and the therapist, resulting in a loss of trust in the interpreter and ruptures in the TA*: “Because the attunement is the basis of the alliance. And with some patients it’s totally fine, but with some patients where the relation is fragile, if they have a lot of mistrust and just one small like-, if you say something where it just sounds like-, you know, your tone of voice is such a big part of how they interpret what is happening, and a small disattunement can make a huge rupture in the relation. So if the relation is difficult already then small ruptures can actually go in and destroy quite a lot in the relation because the patient does not feel like you [the interpreter] take it serious. […] And suddenly you yourself can become a bit frustrated, like, do not drop my alliance on the floor with yawning or with laughing inappropriately.” (IP04)*.

#### 3.2.4. Interpreter as a person taking too much space in the therapeutic room

One aspect, stated by all psychologists, that leads to interpreters being perceived as disruptive to the TA is when they take up too much space in the therapeutic room. This includes the interpreter being emotionally too much involved, showing own emotions in an inappropriate way, giving advice during therapy, comforting or touching the patient, being dressed in a way that attracts a lot of attention, speaking very loud or moving a lot. Most interviewees said that they prefer an interpreter who is barely visible, but at the same time acknowledged that an interpreter changes the relational dynamic just by being physically present. One interviewee described it as disruptive when the interpreter as a person, with his/her personality, is too present*: “And the less the interpreter is there as a person in the room, the more easy it is to do therapy with an interpreter.”*

However, the same psychologist also mentioned at another time of the interview that especially the warm and calm personality of some interpreters was perceived as beneficial for the TA*: “And then I think a lot of interpreters have a very good calm way of being. And that can create a strong alliance, you know, with what they come with. If they come with a warmth and a calmness that can make it actually so you are-, so you become an even stronger alliance all together. “(IP04).*

#### 3.2.5. Interpreters interfering with psychologists’ intention

All psychologists have experienced situations where the interpreter acted in a way that was contrary to the psychologists’ intention or the therapeutic goal. Such contrary and interfering behavior can lead to irritation, frustration and anger on the part of the psychologist. Reasons for such interfering behavior were seen in cultural beliefs (e.g., shame and stigma), a lack of knowledge, a lack of empathy or the fact that patient’s narratives are too terrible and thus unbearable for the interpreter. One interviewee explained that some interpreters tend to avoid the heaviness especially of traumatic narratives by talking faster, changing the tone of their voice, making jokes, laughing and trying to lighten the mood. However, this could perceived by the patient that the TA is not stable enough for their (traumatic) experiences and that it is better not to talk about them*: “They [the interpreters] think sometimes they help the patient, ‘we make it lighter, we need some laugh, we need to have something else because I want to help’. […] It’s avoidance. As a psychologist, I do not want the patient to learn that. As human beings, we have this tendency to go this avoidance path because it’s too hard. And when the patient learns from me or the interpreter that it’s too heavy, you have to do something else to compensate it, then, what is the learning point here? I should not talk about it.” (IP02).*

#### 3.2.6. Dealing with conflicts

All psychologist emphasized the necessity of dealing with conflicts which could lead to ruptures in the TA. However, finding the right time to address such conflicts was described as a fine line. In the case of interpreter misconduct most psychologists prefer to talk to the interpreter in private afterwards. One psychologist explained that dealing with conflicts during the therapeutic session could make the interpreter-therapist alliance too prominent in the therapeutic room, take the focus away from the patient and resulting in an even bigger rupture. Moreover, it was feared that addressing a conflict during the therapy session would lead to the patient no longer perceiving the interpreter and therapist as a team and thus losing trust in both. However, if there is a risk that the conflict will make the patient feel uncomfortable, it should be addressed directly in the therapy session*: “I would probably do it afterwards. Unless it was very obvious in the conversation, and I could see it made the patient distrustful, uncomfortable. Then, I might in a nice way ask, ‚Oh, it seems like she talked for way longer. Would you mind just repeating what she said? ‘Something like that, because if I do not point it out, if it seems like something is wrong, and I do nothing, the patient’s trust in me can also go down, you know, so it can affect the TA.” (IP01).*

Although all participants stressed the importance of clarifying conflicts with the interpreter (during or after the therapy session), there was also a tendency to avoid such confrontation because they were concerned to offend the interpreter and thus jeopardize their working alliance with the interpreter. Criticism was described to be particularly difficult on a personal level, for example when interpreters appear disinterested, yawn or laugh during a conversation. It was described as easier to express criticism on a more technical level, e.g., when the interpretation seems to be incomplete or when interpreter and patient start a conversation during the therapy session*: “But how they present themselves in the room, when you sometimes think that they are not acting very professional, that’s also vulnerable for the interpreter[…]. In those instances I wait until after the session, if I do it at all actually. I would say with those things, with the yawning and laughing, it’s a bit sore. That’s hard to talk about. It feels like-, it can be a bit vulnerable because I’m sure no interpreter does this on purpose, you know.” (IP04).*

#### 3.2.7. Focus on nonverbal communication

Due to the language barrier all psychologist stressed the importance of building an alliance with the patient through nonverbal behavior. This includes for instance the body language (e.g., leaning a bit forward toward the patient), tone variation in the voice, having eye contact with the patient also when the interpreter is talking, active listening (saying “hmm,” nodding), and reacting with facial expressions toward the patient when the interpreter speaks: *“I think it is a lot the nonverbal communication, how important that becomes when it is interpreted. To very much show that I’m focusing on the patient, that I’m not turning towards the interpreter and listening to them, and then I also set an example for the patient. So I want our communication to be direct. And then it goes through a third person, but the alliance is between us, we are in communication.”(IP01).*

To facilitate the development of a good therapist-patient alliance through nonverbal behavior all participants emphasized the seating position and that the patient and therapist should sit facing each other so that they can look each other in the eyes.

### 3.3. Characteristics of trauma-affected refugee patients

#### 3.3.1. High level of distrust as a coping mechanism

All psychologist experienced a high level of distrust among most trauma-affected refugee patients, mainly because of traumatic experiences, especially interpersonal ruptures and breaches of trust, as well as post-migration problems. The distrust was described as a natural and almost necessary coping mechanism which the patients learned over time to survive their traumatic experiences*: “I mean, for example, people who have experienced war, who have been imprisoned, who have experienced genocide and what not. They have really seen the worst of human beings in behavior. And that, in itself, obviously impacts the way you trust other people in general. […] And if your coping mechanism or the way you have been used to-, the way you also take care of yourself is being cautious of others, then that is also in the room obviously.” (IP06).*

#### 3.3.2. Understanding of mental disorders and psychotherapy, cultural beliefs and stigmatization

All psychologists described a lack of knowledge about the concept of psychotherapy as a barrier for building a trusting TA. Patients not only need to trust the therapist, they also need to engage in the method of psychotherapy. The existence of different treatment expectations and explanatory models of mental disorders were described as challenging. The importance of psychoeducation was particularly emphasized. Four psychologists mentioned that especially when the concept of psychotherapy is foreign, patients’ cultural beliefs and fear of stigmatization can be hampering the formation of a good TA. Some patients might feel ashamed to talk to a psychologist and thus hesitant to trust them*: “Because it is so new, it’s so foreign, the idea of psychotherapy, going to see a psychologist, it’s a lot of stigma. So, it takes extra time to kind of get comfortable and create an alliance sometimes, before we can actually start to work. “(IP01).*

#### 3.3.3. Perception of authorities

Four participants pointed out that refugees often have to meet certain immigration requirements in their host country. They explained that psychotherapy could feel like such a requirement and psychologists might be equated with other representatives of authorities whose orders they have to obey. This equation can negatively impact the TA in that patients sometimes do not have good experiences with authorities*: “Some people feel like they have to be here, even though it’s voluntary. And that, of course, can make it hard to create an alliance […]. Often they do not have a very good experience with the government. Sometimes they think we are the government as well. “(IP01).*

## 4. Discussion

This study explored how the presence of interpreters affect the TA in psychotherapy with trauma-affected refugee patients. Semi-structured interviews with psychologists working in an outpatient mental health care clinic in Denmark were conducted.

The presence of interpreters was perceived ambivalently with regards to the TA. On the one hand, therapist expressed great appreciation toward the interpreters and valued their presence as highly beneficial mainly because they enable mutual linguistic understanding between the therapist and patient, a fundamental component of forming a therapeutic alliance. On the other hand, various concerns were expressed, and some therapists seemed to view the interpreter more as a necessary but unstable variable that can impede the formation of a good TA easily.

### 4.1. Interpreter as an (not too) active part of the therapeutic alliance

The interpreter as a person was seen by all participants as an integral and active component of the TA. This is consistent with role conceptualizations that move beyond the conduit role of interpreters in psychotherapy (i.e., interpreters as a non-thinking and non-feeling translation machines (Dysart-Gale, 2005; Hsieh, 2008)) to an understanding of interpreters as an interactive variable of the therapeutic process whose presence changes the therapeutic and relational dynamics (Dysart-Gale, 2005; Miller et al., 2005; Hsieh, 2006, 2008; Hunt and Swartz, 2017; Chang et al., 2021; Delizée and Michaux, 2022). However, viewing the interpreter as an integral and active part of the TA, instead of a mere translation machine, entails increased relational complexity which was experienced as challenging by all participants.

Although, the interpreter was perceived as an active part of the TA, finding the right amount of activeness seems to be challenging. While therapists expressed the preference of an interpreter who is barely visible as a person in the therapeutic room, they also requested that the interpreter is present as a human being and emotionally attuned to what is happening in the therapeutic room. A lack of emotional attunement as well as being too emotionally involved was perceived as hampering the formation of the TA. Authors, like Angelelli (2004) and Gryesten et al. (2021) discuss that while interpreters’ visibility is usually defined as something categorial (visible vs. invisible), it seems to be more a flexible continuum of visibility in reality, depended on, for instance, the therapeutic stage and content. Also, based on the examples described by the participants in this study, it appears that the visibility of the interpreter is not categorical, but should vary depending on the situation.

### 4.2. Balancing the therapist-interpreter-patient alliance triangle

Based on the study findings the TA in IMPs can be described as a therapist-interpreter-patient alliance triangle consisting of three distinct alliances which were perceived as different but strongly intertwined and mutually influential: the therapist-patient, therapist-interpreter and interpreter-patient alliance. In line, Delizée and Michaux (2022) elaborate that by actively forming an alliance with the therapist and the patient, the interpreter might indirectly influence the alliance formation between therapist and patient. Participants in this study stated that forming and maintaining a good TA requires balancing all these three dyadic alliances, whereby forming a trustful therapist-patient alliance is still viewed as the main focus. Referring to Bordin (1979) there needs to be a personal bond and an agreement on the therapeutic tasks and goals between the three of them.

Previous authors described a good alliance triangle as dynamic with constantly changing distances between the parties involved depending on the therapeutic stage and content (Miller et al., 2005; Tribe and Thompson, 2009a; Hsieh and Hong, 2010). Also, participants in this study experienced shifting and changing relational dynamics. For instance, at the beginning of the therapeutic process, some patients might be closer to the interpreter due to the cultural and linguistical proximity. As the therapy progresses patients’ primary orientation (ideally) shifts from the interpreter to the therapist. However, if one of the dyadic alliances is consistently either too strong (meaning too close) or too weak (meaning too distant), or if ruptures or conflicts occur, the alliance triangle can become unbalanced. As reported in other interview studies with therapists (Raval and Smith, 2003; Miller et al., 2005; Hanft-Robert et al., 2018), participants in this study expressed feelings of distance, being excluded, powerless, and incompetence resulting in disengagement from the TA, if they perceive that the patient is solely orientated toward the interpreter. In such cases, it was described as helpful if the interpreter uses subtle gestures or facial expressions to direct the patient slowly toward the therapist during the course of therapy.

### 4.3. Forming a good therapist-interpreter working alliance

Keeping the alliance triangle in balance requires the establishment of a good working alliance between therapist and interpreter. Participants described it as challenging to find the right balance between a friendship-like closeness and professional distance with the interpreter. Gryesten et al. (2021) stated that the collaboration between interpreters and psychologists can range from a friendship to a collegial to an employer-employee alliance. Raval and Smith (2003) noted that the therapist-interpreter working alliance differs greatly compared to the alliance between two therapists especially due to potential power and hierarchical differentials. A lack of professional recognition can place the interpreters in an unequal, less powerful position. Moreover, the interpreter perceiving the therapist as a White professional authority figure can affect the working alliance between them (Raval and Smith, 2003). Also, therapists in this study expressed that a good working alliance requires mutual appreciation of each other’s profession and emphasized that therapists need to acknowledge interpreters as professionals. Delizée and Michaux (2022) added that the interpreter needs to understand the goals and methods of psychotherapy and that besides mutual trust and respect, a clear understanding of roles is necessary for alliance formation. This highlights why the use of untrained individuals as interpreters (e.g., family member, multilingual staff members) should be avoided and the use of qualified or so called professional interpreters encouraged (Flores, 2005; Karliner et al., 2007; Flores et al., 2012; Kilian et al., 2014).

### 4.4. Changes in the therapist-patient alliance

On the one hand, participants strongly emphasized that the interpreter can facilitate the formation of an trustful therapist-patient alliance. Patients tend to perceive the interpreter, who is often someone who shares the same cultural backgrounds as the patient, as a confidant and holding a safe space in an often unfamiliar and unsettling environment. Especially in early stages of the therapy and when the concept of psychotherapy is new to some of the patients, the presence of an interpreter can demonstrate and encourage that the therapist and the method of psychotherapy are trustworthy. Similar, therapists in other studies reported that the presence of an interpreter can enhance the feeling of trust and security in patients, which in turn is seen as a foundation to form the therapist-patient alliance (Delizée and Michaux, 2022). On the other hand, interpreters were perceived as a filter between the therapist and the patient. The interpreter is automatically the first person to hear and react to what the patient has said. The therapist receives the linguistic information with a delay, which can lead to a sense of distance, emotional misattunement and less intimacy in the therapist-patient alliance, especially for therapists who are inexperienced in working with interpreters. In line, Raval and Smith (2003) found that therapists feel shielded and losing the emotional context due to the translation process. Also other studies have shown that while both therapists and patients consider the involvement of interpreters as the best alternative to no treatment, they prefer a dyadic setting and especially therapists perceive the interpreters’ presence as disruptive for developing rapport with patients (Raval and Smith, 2003; Miller et al., 2005; Villalobos et al., 2016; Hanft-Robert et al., 2021).

### 4.5. Dealing with distrust, conflicts, and ruptures in the alliance

It is known that patients’ distrust toward the interpreter can negatively affect the therapeutic process (Hunt and Swartz, 2017; Dabic, 2021; Hanft-Robert et al., 2021; Delizée and Michaux, 2022). Also, participants in this study emphasized that patients’ distrust, arising for example from doubts about the interpreter’s professionalism and commitment to confidentiality, hinders the formation of a good TA. The interpreter being continuously present, being impartial, having sufficient language skills, interpreting precisely and completely can help to strengthen the patient-interpreter alliance. Before the actual therapeutic work starts, the role of the interpreter and their obligation to adhere to professional ethical principles, such as confidentiality, should be explained to the patient. Moreover, interpreters’ behavior that interferes with the therapists’ intention, such as joking or laughing to lightening the atmosphere, can lead to frustration and ruptures especially in the working alliance between interpreter and therapist. However, having a strong personal bond can prevent that interpreters’ misconduct leads to ruptures in the TA.

The therapists described that distrust, ruptures and conflicts in one of the dyadic alliances can also be transferred to one of the other alliances. For example, if the interpreter laughs about something the patient has said, this could lead to a rupture in the patient-interpreter alliance. However, such ruptures are happening in the therapeutic room, for which the therapist holds the responsibility. If they are not noticed and not handled appropriately by the therapist, they will also affect the quality of the patient-therapist alliance. Thus, therapists described it as crucial not only to ensure the quality of their respective alliances with the patient and the interpreter, but also to be continuously attentive to (ruptures in) the interpreter-patient alliance. Similar results can be found in other therapeutic settings where more than two persons are involved, such as couple and family therapies. Dyadic yet mutually interdependent alliances exist, thus ruptures in one dyad can be transferred to the other (Rait, 2000).

Participants stated various factors that can jeopardize the alliance formation. However, dealing appropriately with distrust, conflicts or ruptures seems to be challenging. Therapists expresses uncertainty when and how to address conflicts with the interpreter. They feared that addressing a conflict in front of the patient could make the interpreter-therapist alliance too prominent in the therapeutic room. However, if the conflict affects the therapeutic process or makes the patient feel uncomfortable, it should be addresses immediately. A tendency of avoiding confrontation with the interpreter was shown, especially when a conflict or misbehavior appears on a personal level, e.g., if the interpreter seems to be bored. In line, Gryesten et al. (2021) found on part of therapists an inherent conflict between maintaining a friendly and collegial alliance with the interpreter and their responsibility for the therapeutic process.

### 4.6. Impact of trauma and flight on the therapeutic alliance

The findings revealed that besides the presence of an interpreter, specific characteristics of trauma-affected refugees can affect the formation of the TA. However, it should be noted that these characteristics can also impact the TA formation when working with migrant patients in general. Firstly, it was emphasized that traumatic experiences, especially interpersonal breaches of trust, can lead to a high level of general interpersonal distrust as a form of coping mechanism, which can hamper the formation of a trusting TA. Morina et al. (2016) found that interpersonal trauma experiences, such as torture, are associated with more avoidant attachment tendencies in refugees. In turn, it could be shown that patients with an avoidant attachment style have more difficulties in establishing and maintaining a good TA (Smith et al., 2010). The therapist needs to be aware that a possible high level of general distrust functioning as a coping mechanism can prolong the formation of a TA. Therapeutic interventions focusing on the establishment of a stable alliance at the beginning of the therapy seem to be particularly significant when working with trauma-affected refugee patients.

Secondly, and in line with previous findings (Sandhu et al., 2013; Byrow et al., 2019; Satinsky et al., 2019; Hanft-Robert et al., 2021), participants perceived patients’ unfamiliarity with the concept of psychotherapy as well as possible stigmatization of mental disorders and psychotherapy as challenging for the TA formation. It is well-investigated that in psychotherapy with migrants and refugees, both the professional as well as the patient are shaped by their cultures, and that their treatment expectations can differ greatly due to culturally based beliefs (Sandhu et al., 2013). According to Bordin (1979), therapist and patient, however, need to have an agreement on the therapeutic goals and tasks in order to form a good alliance, which requires a shared understanding of the therapeutic concept and treatment process between therapist and patient.

Thirdly, the therapists stated that they might be equated by refugee patients with representatives of other, often negatively associated, authorities, which can hinder the development of a trusting personal bond, which is the foundation of any good TA according to Bordin (1979). Similarly, Sandhu et al. (2013), who interviewed mental health care professionals in 16 European countries, reported that the development of trust with migrant patients can be affected by a general distrust to any form of authority or public service stemming from negative experiences, such as torture, oppression and ethnic conflicts (Sandhu et al., 2013).

Based on these study findings, preparatory sessions focusing on cultural-sensitive psychoeducation might be useful before beginning the actual therapeutic process when working with trauma-affected refugee patients. These preparatory sessions should include an explanation of the framework of psychotherapy and the clarification of the therapists’ role, especially in distinction to other institutions and authorities. Moreover, different treatment expectations, cultural beliefs and explanatory modules of mental disorders should be explored and a shared understanding of the following psychotherapeutic process developed.

### 4.7. Strengths and limitations of the study

When interpreting the study findings some limitations must be taken into account. First, due to the relatively small sample size data saturation cannot be ensured. However, all possible participants at the specific clinic were invited to participate. Secondly, the study participants were recruited only from one outpatient clinic that is specialized in mental health care for migrant and refugee patients with trauma-related mental health problems. Thus, most of the therapists were experienced in working with trauma-affected refugees and interpreters. To increase the variation in results, it would be useful to interview therapists from multiple inpatient and outpatient clinics, and especially include less experienced therapists. It must also be critically noted that only one male participants could be included in this study. This may have shaped our findings, as gender seems to be a relevant factor for the TA formation (Wintersteen et al., 2005). Moreover, most participants were trained in CBT. Further studies should include different therapeutic orientations. Even though the TA can be considered as a common factor in psychotherapy, there can be differences in how the alliances are formed. One inclusion criteria in this study was that the interviewed therapists have completed at least one course of IMP, which is a minimum of 10 therapy sessions. Since the duration of therapy can have an influence on the quality of the TA (Sharf et al., 2010; Mander et al., 2013), it would be useful to focus in future studies on how the TA in IMP changes over time, e.g., by assessing the quality of TA at different time points during the therapeutic process. Furthermore, the interviews were conducted in English as participants second language. However, this does not seem to have resulted in linguistic limitations.

The greatest strength of this study is that it focuses exclusively on the TA formation. Most of the studies to date focus more on general benefits and challenges of working with interpreters and the successful collaboration between interpreter and therapist. With the exception of gender and therapeutic orientation, the sample was as heterogeneous as possible in terms of, for example, professional experience and ethnicity. Further studies should include interpreters as well as patients and include different methods, e.g., quantitative measurement of the quality of the TA or observation. In addition, it would be interesting to focus more on cross-racial aspects and how the interplay of different ethnic and cultural backgrounds of therapists, interpreters and patients affect the TA in IMP.

## 5. Conclusion

The presence of an interpreter on the TA formation in therapy with trauma-affected refugees was perceived ambivalently. It became clear that the interpreter is an active part of the TA, although there were contradictory perceptions regarding the amount of activeness and personal representation on part of interpreter. An alliance triangle was described, consisting of different but intertwined and mutually influencing dyadic alliances. Compared to other settings where the interpreter’s skills are of greater importance for the quality of the conversation (e.g., conference interpreting), in psychotherapy the formation of a good TA between all parties involved seem to be most relevant. However, building a good TA in IMP seems to be complex and an act of balance depending on various factors. The study results and derived recommendations can be used to develop guidelines or training for IMP.

## Data availability statement

The datasets presented in this article are not readily available because public availability of data could potentially compromise participant privacy. Participants did not consent to have their full transcripts made publically available. Requests to access the datasets should be directed to s.hanft-robert@uke.de.

## Ethics statement

Ethical review and approval was not required for the study on human participants in accordance with the local legislation and institutional requirements. The patients/participants provided their written informed consent to participate in this study.

## Author contributions

SH-R, LGL, JC, and MM contributed to conception and design of the study. SH-R collected the data, analyzed the data with support of LGL, and wrote the first draft of the manuscript. SH-R, LGL, JC, and MM discussed the results. LGL, JC, and MM read the manuscript several times and provided significant feedback. All authors contributed to manuscript revision, read, and approved the submitted version.

## Funding

The authors acknowledge financial support from the Open Access Publication Fund of UKE - Universitätsklinikum Hamburg-Eppendorf- and DFG – German Research Foundation.

## Conflict of interest

The authors declare that the research was conducted in the absence of any commercial or financial relationships that could be construed as a potential conflict of interest.

## Publisher’s note

All claims expressed in this article are solely those of the authors and do not necessarily represent those of their affiliated organizations, or those of the publisher, the editors and the reviewers. Any product that may be evaluated in this article, or claim that may be made by its manufacturer, is not guaranteed or endorsed by the publisher.

## Footnotes

^1^More information: http://en.nvk.dk/how-to-notify/what-to-notify
