# Supplementary material for: A balancing act: how interpreters affect the therapeutic alliance in psychotherapy with trauma-affected refugees—a qualitative study with therapists
Source: Front Psychol. 2023 May 16;14:1175597. doi: 10.3389/fpsyg.2023.1175597 (PMC10228651; doi:10.3389/fpsyg.2023.1175597)
Supplement: Supplementary file 1 [file Data_Sheet_1.PDF]

UKE
HAMBURG

Interview guide

	Questions	Notes
	At the beginning, I would like to talk about the therapeutic alliance in general, when no interpreter is involved.	
1	From your point of view, how **important** is a good therapeutic alliance between you as a psychologist and the patient? Why?<br>a) At CTP you often work with trauma-affected refugee patient. To what extent is a good therapeutic alliance particularly important in this group of patients? Why?	
2	Could you tell me a little about **how** a good therapeutic alliance between you and the patient can be **build**?<br>a) Can you give me some examples of what you have done in previous therapies to build a good alliance with the patient?<br>b) How did you know you and the patient had a good alliance?<br>c) What are special aspects or challenges in building a good therapeutic alliance with trauma-affected refugee patients?	
	When conducting interpreter-mediated psychotherapies, it is no longer just you and the patient who are present. There is another third person, the interpreter.	
3	From your point of view, what **changes** in the alliance between you and the patient have you experienced due to the presence of an interpreter?<br>a) You mentioned you do *XY* to build a good therapeutic alliance with the patient. What effect does the presence of an interpreter have on that?<br>b) You said that you can tell from *XY* that you and the patient have a good alliance. What effect does the presence of an interpreter have on that?	
4	Can you give me an example where you perceived the presence of the interpreter affected the alliance between you and the patient **negatively**?<br>a) Which specific characteristics or behaviours of the interpreter did you experience as challenging? Why?<br>b) If you try to put yourself in the patient's shoes. What might make it difficult for patients, especially trauma-affected refugee patients, to form a good alliance with you when an interpreter is present?	
5	So, there might be some challenges on the one hand. On the other hand, can you give me an example where you perceived the presence of the interpreter affected the alliance between you and the patient **positively**?<br>a) Which specific characteristics or behaviours of the interpreter did you experience as helpful? Why?<br>b) If you try to put yourself in the patient's shoes. What might make it easier for patients, especially trauma-affected refugee patients, to form a good alliance with you when an interpreter is present?	

6	If you think about your current or most recent interpreter-mediated therapy. How would you say the therapeutic alliance between you and the patient has **changed or developed** throughout the therapy? Why?	
7	When building a good therapeutic alliance, **empathy**, meaning the ability to understand another's personal experiences, to show the other person that you understand and to act in a helpful manner, is particularly important. What difficulties do you experience regarding empathic communication in interpreter-mediated therapies with trauma-affected refugee patients?<br>a) How do you deal with these difficulties?<br>b) Are there any benefits that you experience? Which ones?	
8	Another aspect of a good therapeutic alliance is mutual **trust**. What challenges do you experience regarding building trust in interpreter-mediated psychotherapy with trauma-affected refugee patients?<br>a) How do you deal with these difficulties?<br>b) Are there any benefits that you experience? Which ones?	
9	In interpreter-mediated psychotherapy, there is you and there is the interpreter. Two "professionals", so to speak. To what extent did you ever feel that the interpreter and you were somehow **competing with each other**? *(e.g. competing for the patient's favor, expertise, control,…).*<br>a) What was the issue? Could you describe the situation?<br>b) Why was it problematic from your perspective?<br>c) How did you deal with it?	
10	We have talked a lot about specific aspects regarding the therapeutic alliance in interpreter-mediated psychotherapy. If you think about all of your current and past interpreter-mediated therapies. What would you say you have **learned** over time about how to create and maintain a good therapeutic alliance between you and the patient with an interpreter present?	
	We are now at the end of the interview. Is there anything else we have not talked about, but which is important from your point of view?	
	Lastly, I have a few questions about you as a person and a psychotherapist (short questionnaire on sociodemografic data).	

Short questionnaire on sociodemografic data

0	Participants' code:						
	1, 2: first two letters of your mother's first name<br>3, 4: first two letters of your favorite colour<br>5, 6: day of the interview	1	2	3	4	5	6

1 To which gender identity do you most identify?

☐ female ☐ male ☐ other ☐ prefere not to say

2 How old are you?

.... years

3 Did you or your parents immigrate to Denmark?

☐ yes, myself from: ________________________________ (country of birth)

☐ yes, my mother from: ________________________________ (country of birth)

☐ yes, my father from: ________________________________ (country of birth

☐ no

4 Are you a licensed psychologist?

☐ yes, since: .... (year)
☐ no

5 For which therapeutic approach are you trained?

☐ CBT ☐ Psychoanalysis ☐ Systemic therapy
☐ Psychodynamic therapy ☐ Humanistic therapy ☐ Other: ....

6 How many years of experience do you have as a psychologist?

.... (years)

7 How many years of experience do you have in psychotherapy with trauma-affected refugee patients?

.... (years)

8 How many years of experience do you have in conducting interpreter-mediated psychotherapy?

.... (years)

9 How often do you conduct interpreter-mediated psychotherapies (last 6 months)?

.... (times per ☐ week or ☐ month)

10 Did you receive extra training for conducting interpreter-mediated psychotherapy?

☐ yes
☐ no

11 What kind of training?

11.1 When and how many hours in total?

.... (year) .... (hours)

Thank you very much for your time and the interesting and enriching interview!
